# Alcohol Use Disorder—Stress, Sense of Coherence, and Its Impact on Satisfaction with Life

**DOI:** 10.3390/jcm14176183

**Published:** 2025-09-01

**Authors:** Monika Pajewska, Olga Partyka, Aleksandra Czerw, Katarzyna Sygit, Paulina Wojtyła-Buciora, Sławomir Porada, Izabela Gąska, Magdalena Konieczny, Elżbieta Grochans, Anna Maria Cybulska, Daria Schneider-Matyka, Ewa Bandurska, Weronika Ciećko, Jarosław Drobnik, Piotr Pobrotyn, Dorota Waśko-Czopnik, Julia Pobrotyn, Adam Wiatkowski, Łukasz Strzępek, Michał Marczak, Tomasz Czapla, Remigiusz Kozlowski

**Affiliations:** 1 Department of Health Economics and Insurance, Center for the Humanities and Social Sciences of Medicine, Medical University of Warsaw, 00-581 Warsaw, Poland; 2 Department of Economic and System Analyses, National Institute of Public Health NIH-National Research Institute, 00-791 Warsaw, Poland; 3 Faculty of Medicine and Health Sciences, University of Kalisz, 62-800 Kalisz, Poland; 4 Faculty of Health Sciences and Psychology, Collegium Medicum, University of Rzeszów, 35-310 Rzeszow, Poland; 5 Medical Institute, Jan Grodek State University in Sanok, 38-500 Sanok, Poland; 6 Department of Nursing, Faculty of Health Sciences, Pomeranian Medical University in Szczecin, 71-210 Szczecin, Polandanna.cybulska@pum.edu.pl (A.M.C.);; 7 Center for Competence Development, Integrated Care and e-Health, Medical University of Gdansk, 80-204 Gdansk, Poland; 8 Department of Family Medicine, Faculty of Medicine, Wroclaw Medical University, 51-141 Wroclaw, Poland; 9 Pulsantis Specialist and Rehabilitation Clinic Ltd., 53-238 Wroclaw, Poland; 10 Department of Gastroenterology, Hepatology with Inflammatory Bowel Disease Subunit, Provincial Specialist Hospital J. Gromkowskiego, 51-149 Wroclaw, Poland; dczopnik@gmail.com; 11 Faculty of Medicine, Wroclaw Medical University, 50-345 Wroclaw, Polandadam.wiatkowski@student.umw.edu.pl (A.W.); 12 Clinical Department of General and Oncological Surgery, Saint Raphael Hospital, 30-693 Cracow, Poland; 13 Department of Surgery, Andrzej Frycz Modrzewski Cracow University, 30-705 Cracow, Poland; 14 Department of Innovation of Merito University in Poznan, 61-895 Poznan, Poland; 15 Department of Management, Faculty of Management, University of Lodz, 90-237 Lodz, Poland; tomasz.czapla@uni.lodz.pl; 16 Department of Management and Logistics in Healthcare, Medical University of Lodz, 90-131 Lodz, Poland; remigiusz.kozlowski@umed.lodz.pl

**Keywords:** alcohol addiction, sense of coherence, SOC, stress

## Abstract

**Background:** Alcohol use disorder (AUD) is a chronic relapsing brain disorder characterized by compulsive alcohol seeking, loss of control over drinking, and negative emotional states when not using. It has significant psychological, physiological, and social consequences, often co-occurring with mental health disorders such as depression and anxiety. Psychological resilience is gaining more recognition. Sense of coherence (SOC) could be treated as a health factor, and individual predispositions play a crucial role in fighting disease and addiction. Our study examines whether SOC and its components—comprehensibility, manageability, and meaningfulness—predict life satisfaction in patients with AUD and whether perceived stress and health behaviors mediate these relationships. **Methods:** The study was conducted on a sample of 100 adult patients diagnosed with alcohol use disorder. **Results:** We found that the higher the manageability and meaningfulness, the lower the level of perceived stress and the higher the level of preventive behavior. Notably, perceived stress emerged as a significant mediator between SOC and satisfaction with life, while health behaviors did not show a mediating effect. **Conclusions:** The findings emphasize the protective role of SOC in enhancing psychological well-being among individuals with AUD and suggest that interventions aimed at strengthening SOC may reduce stress and improve overall life satisfaction in this population.

## 1. Introduction

In recent years, the significance of psychological resilience has gained increasing attention in the field of health psychology, particularly among individuals struggling with addiction [1,2]. Sense of coherence (SOC), a concept created by Aaron Antonovsky, could be treated as a health factor, and individual predispositions play a crucial role in fighting disease and addiction [3]. Sense of coherence may be defined as an “ability to comprehend the situation, and the capacity to use the resources available”. It is related to a person’s perception and their ability to cope with stressful situations and to use available resources [3]. Therefore, there is an association between sense of coherence and perceived stress. There is also a link between a higher sense of coherence and the ability to avoid risky health behavior, including the use of alcohol or smoking [4]. The relationships between sense of coherence and health behaviors were found in empirical studies [5,6]. Sense of coherence and perceived stress were found to be related to acceptance of illness in an empirical study conducted on a group of patients undergoing alcohol addiction therapy [7]. Sense of coherence and perceived stress were also found to be related to satisfaction with life in an empirical study conducted on a group of patients with diagnosed alcohol use disorder [8]. The level of stress and health behaviors are related to satisfaction with life [9]. Coping mechanisms are the strategies individuals employ to manage stress and emotional tension [10]. These mechanisms can significantly influence the trajectory of addiction and recovery [11].

According to the WHO, alcohol use disorder affects 1 in 11 adults [12]. The WHO European Region has the highest alcohol consumption and the greatest burden of alcohol-related harm in the world [13]. Europe remains the heaviest drinking region globally, with an average yearly per capita consumption of 11 L. It is estimated that 1 in 4 deaths in the age group 20–24 is related to alcohol consumption [13]. According to the data provided by Eurostat in 2021, household expenditure on alcoholic beverages in the European Union countries amounted to EUR 128 billion [8]. Among the EU member states, the highest percentage of expenditure on alcoholic beverages was recorded in Latvia (5%), Estonia (4.7%), and Poland (3.7%) [14].

One of the main symptoms of alcohol dependence syndrome is constituted by alcohol craving [9]. Alcohol dependence can also be defined as a condition characterized by an impaired ability to stop or control alcohol use despite adverse social, occupational, or health consequences [15,16,17]. Current epidemiological data support a link between stress and alcohol use disorders [18,19]. However, the connection is not predictably causal. Stress under all circumstances does not necessarily lead to alcohol consumption. Genetic factors and past history of life experiences can influence this interaction. This shows a more complicated relationship between stress and alcohol consumption. Therefore, more research is needed to establish a clearer understanding of this relationship [20].

This study focuses on research questions regarding the interplay between SOC and its associated factors, exploring whether perceived stress and health behaviors serve as mediators in these relationships. Employing a robust methodological approach, the research analyzes data from a diverse sample of individuals aged 18 to 76 who are struggling with alcohol addiction.

## 2. Materials and Methods

Based on the theoretical framework of SOC, which encompasses comprehensibility, manageability, and meaningfulness, our paper aims to illustrate how these dimensions influence mental health outcomes and health-related behaviors.

Our study was cross-sectional. The participants completed five questionnaires: SOC-29, PSS-10, AIS, HBI, and SWLS. SOC-29 was used to measure sense of coherence [3]. The questionnaire consists of 29 questions, with each scored from 1 to 7. The sum of scores for the answers gives a total score within the range of 29–203 points. The higher the score, the higher the sense of coherence. However, a sense of coherence is considered to be a multidimensional construct. Therefore, in addition to the total score, the questionnaire also allows for calculating scores on three subscales, namely, comprehensibility (the cognitive aspect of sense of coherence), manageability (the instrumental dimension), and meaningfulness (the motivational dimension). The scores for the three subscales are based on 11 items, 10 items, and 8 items, respectively.

The PSS-10 (Perceived Stress Scale) questionnaire was used for measuring the intensity of experienced stress [20,21]. The questionnaire consists of 10 questions, each scored from 0 to 4. The sum of scores for the answers gives a total score within the range of 0–40 points. The higher the score, the higher the level of experienced stress.

For the Acceptance of Illness Scale (AIS), the questionnaire was used to assess the degree of acceptance of illness by the patient. The AIS tool consists of eight statements addressing the negative effects of poor health, such as difficulties in adapting to the limitations resulting from the illness, changes in self-esteem, or feelings of uselessness [21].

Zygfryd Juczyński’s “Health Behaviour Inventory” (HBI) was used to evaluate pro-health behaviors undertaken by patients. HBI consists of 24 statements relating to various types of positive pro-health behaviors. The higher the score, the greater the intensity of the declared pro-health behaviors. The inventory includes four subscales examining proper eating habits, preventive behavior, and positive mental attitude [21].

The SWLS (Satisfaction with Life Scale) questionnaire was used for measuring life satisfaction [21]. The questionnaire consists of 5 questions, each scored from 1 to 7. The sum of scores for the answers gives a total score within the range of 5–35 points. The higher the score, the higher the level of life satisfaction.

In the current study, we aimed at:

1. Analyzing the relationships between the sense of coherence and its dimensions and the level of perceived stress, the level of health behaviors, the level of acceptance of illness, and satisfaction with life while controlling for participants’ age;
2. To determine if the level of perceived stress and health behaviors were parallel mediators of the relationships between the sense of coherence and both satisfaction with life and acceptance of illness, while controlling for participants’ age.

First, descriptive statistics for the analyzed interval variables were calculated to assess their distributions. Next, the correlation analysis between all analyzed variables was performed. In the main part, linear regression models in which the indicators of the sense of coherence were analyzed as predictors of satisfaction with life, perceived stress, health behaviors, and acceptance of illness. Because the indicators of the sense of coherence are positively related to each other, Variance Inflation Factor values were calculated to verify if collinearity biases the results acquired. Finally, a path analysis was performed to assess if the level of perceived stress and health behaviors were parallel mediators of the relationships between the sense of coherence and both satisfaction with life and acceptance of illness. We evaluated the model fit with the use of fit indices, i.e., CFI, NFI, SRMR, and RMSEA. The majority of calculations were performed with the use of IBM SPSS Statistics 30.0. Path analysis was performed in Jamovi 2.6.26 software.

### Descriptive Statistics

The study was conducted on a sample of 100 patients diagnosed with alcohol use disorder aged 18–76 (M = 42.07; SD = 12.11), with 66 males aged 21–76 (M = 43.24; SD = 11.77) and 34 females aged 18–66 (M = 39.79; SD = 12.62). The following sample selection criteria were used: persons over 18 years of age, without any gender restrictions, and undergoing treatment in a psychoactive substance addiction treatment ward or an addiction treatment ward or in an outpatient addiction therapy clinic as part of services financed from public funds.

Table 1 presents descriptive statistics for the analyzed interval variables, i.e., the mean values, standard deviations, minimum values, maximum values, and measures of skewness and kurtosis. Also, Pearson correlation coefficients between the analyzed variables are provided.

The distributions of analyzed variables did not deviate significantly from the normal distribution, with the exception of correct eating habits and acceptance of illness. However, the measures of skewness and kurtosis for both these variables were in the range of [−1; 1]. Table 2 below presents Pearson correlation coefficients between the analyzed variables.

All dimensions of sense of coherence correlated positively with each other. These correlations were moderate. Also, all scales for measuring health behaviors correlated positively with each other. These correlations were moderate as well. The age of participants correlated positively with all scales for measuring health behaviors and with comprehensibility. However, these correlations were weak. Satisfaction with life correlated positively with all dimensions of sense of coherence moderately and with all scales for measuring health behaviors weakly, and also moderately and negatively with perceived stress. All dimensions of sense of coherence correlated moderately and negatively with perceived stress and positively with positive mental attitude (moderately), correct eating habits (weakly), and with health behaviors (weakly regarding manageability and moderately regarding other dimensions). Comprehensibility, meaningfulness, and general sense of coherence correlated positively with preventive behavior and with health practices. However, these correlations were weak. General sense of coherence also correlated weakly and positively with acceptance of illness. The level of perceived stress also correlated negatively with all scales for measuring health behaviors (moderately with positive mental attitude and general score for health behaviors, weakly with other health behaviors indicators) and with acceptance of illness (weakly).

## 3. Results

### 3.1. Sense of Coherence as a Predictor of Perceived Stress, Health Behaviors, Acceptance of Illness, and Satisfaction with Life

Comprehensibility, manageability, and meaningfulness were analyzed as predictors of perceived stress, health behaviors, acceptance of illness, and satisfaction with life. Each dependent variable was analyzed in a separate model. Next, the general sense of coherence was analyzed as a predictor of the same dependent variables. All analyses were performed while controlling for the participants’ ages. We performed hierarchical regression analysis in which participants’ ages were included in the first block and sense of coherence indicators in the second block. This applies to each regression analysis for every dependent variable. We performed these analyses twice. First, this included comprehensibility, manageability, and meaningfulness in the second block. Next, this included general sense of coherence in the second block instead of comprehensibility, manageability, and meaningfulness, which are the components of the general sense of coherence. The results for the analyses involving comprehensibility, manageability, and meaningfulness are depicted in Table 3.

None of the variance inflation factors were higher than five, which means that the results were not biased by collinearity. Manageability and meaningfulness were predictors of the level of perceived stress and the level of preventive behavior. The higher the manageability and meaningfulness, the lower the level of perceived stress and the higher the level of preventive behavior. Meaningfulness was the only statistically significant predictor of positive mental attitude, health behaviors, and satisfaction with life. It was positively related to all three. Comprehensibility was the only statistically significant predictor of correct eating habits and health practices. It was positively related to both. The dimensions of sense of coherence were not related to acceptance of illness. The results for analyses involving the general sense of coherence are depicted in Table 4.

The general sense of coherence was a statistically significant predictor of all analyzed dependent variables. It was related negatively to the level of perceived stress and positively to all health behaviors, acceptance of illness, and satisfaction with life. The sense of coherence explained most of the variance of perceived stress (34.1%) and positive mental attitude (30.6%). The weakest associations were detected for preventive behavior (9.1% variance explained and for acceptance of illness (4.3%) of variance).

### 3.2. Perceived Stress and Health Behaviors as Mediators of the Relationships Between the Sense of Coherence and Satisfaction with Life or Satisfaction with Life

**Figure 1:**
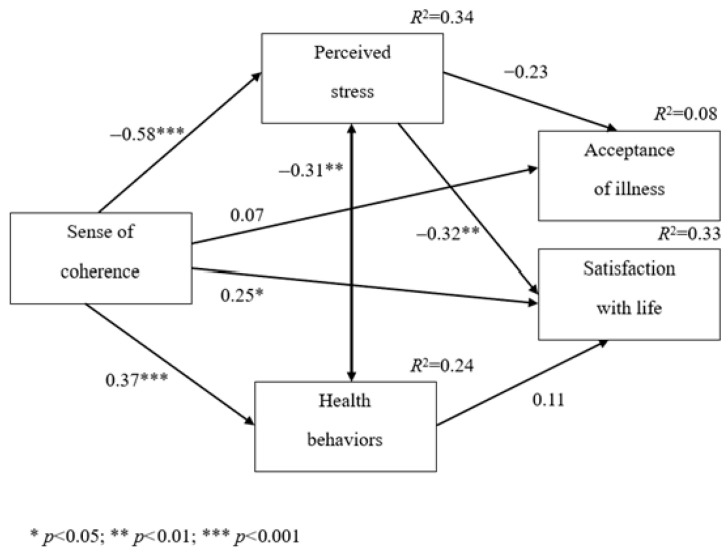
Diagram of the analyzed relationships between variables.

Perceived stress and health behaviors were also analyzed as mediators of the relationships between the sense of coherence and satisfaction with life or satisfaction with life. The diagram of the analyzed relationships between the analyzed variables is depicted in Figure 1.

The analysis was performed with the use of path analysis based on the maximum likelihood method while controlling for the participants’ ages. The results are depicted in Table 5.

The fit indices indicated adequate fit of the analyzed model to the data. They were equal to CFI = 0.99, NFI = 0.99, SRMR = 0.02, and RMSEA = 0.01. The mediation effect in which the level of perceived stress was a mediator of the relationship between the sense of coherence and satisfaction with life was statistically significant, Beta = 0.19, *p* < 0.01. The higher the sense of coherence, the lower the level of perceived stress, and as a consequence, the higher the level of satisfaction with life. However, the level of perceived stress was not a statistically significant mediator of the relationship between the sense of coherence and acceptance of illness, Beta = 0.13, *p* > 0.05, due to the lack of a statistically significant relationship between the level of perceived stress and acceptance of illness. The sense of coherence was positively related to health behaviors, but the level of health behaviors was not a statistically significant mediator of the relationship between the sense of coherence and satisfaction with life, Beta = 0.04, *p* > 0.05, due to the lack of a statistically significant relationship between the level of health behaviors and satisfaction with life. The sense of coherence was also related directly and positively to satisfaction with life, but not to the acceptance of illness. The analyzed model explained 33.2% of the satisfaction with life variance and 7.5% of acceptance of the illness variance, and also 33.6% of the perceived stress variance and 24.1% of the health behavior variance.

## 4. Discussion

Our study focused on examining the role of sense of coherence in predicting perceived stress, health behaviors, acceptance of illness, and satisfaction with life among adult participants, while controlling for age. Consistent with Antonovsky’s salutogenic model, SOC emerged as a robust correlate of both psychological and behavioral outcomes [3]. We tried to interpret these findings in light of the existing literature and suggest directions for future research and practice.

Our results demonstrated that a higher SOC—whether measured as a general composite or its individual dimensions (comprehensibility, manageability, and meaningfulness)—was associated with lower perceived stress. In particular, manageability and meaningfulness were significant predictors of reduced stress (Table 3), and the general SOC accounted for 34.1% of the variance in stress (Table 4). These findings replicate the prior research showing that individuals who view life as structured and meaningful cope more effectively with stressors [22,23]. The mediation analysis further confirmed that perceived stress partially mediates the SOC–satisfaction with life link (β = 0.19, *p* < 0.01), supporting the view that SOC buffers stress to enhance well-being [24,25]. The findings reveal significant correlations among the dimensions of SOC, health behaviors, and perceived stress, highlighting the predictive power of SOC in determining life satisfaction and mental well-being. The relationship between stress levels and alcohol consumption was one of the first areas of research related to alcohol addiction [26]. Stress is a recognized factor in relapse and is also a significant factor in increasing the tendency to drink [27].

Interestingly, while comprehensibility was predictive of certain health practices, it did not significantly correlate with overall life satisfaction. This finding may reflect the complex interplay of factors influencing health behaviors and life satisfaction, indicating that enhancing comprehensibility alone may not suffice in improving overall well-being.

## 5. Conclusions

Our findings highlight the importance of SOC in the context of alcohol addiction and suggest that fostering a strong sense of coherence could serve as a valuable strategy in addiction treatment and prevention. A higher sense of coherence was associated with a lower level of perceived stress, which plays a significant role in the treatment of addiction. Our study was cross-sectional. Therefore, further research, particularly prospective studies, is necessary to explore the mechanisms through which SOC influences coping and recovery outcomes, as well as the potential for developing targeted interventions that enhance SOC among individuals at risk of or recovering from alcohol addiction.

## Figures and Tables

**Table 1:** Descriptive statistics for analyzed interval variables.

Variables	M	SD	Min	Max	S	K	S-W	*p*
Satisfaction with life	17.10	5.61	5	33	0.32	0.10	0.98	0.133
Comprehensibility	40.15	8.55	20	60	0.04	−0.22	0.99	0.650
Manageability	43.29	7.86	19	61	−0.10	0.07	0.99	0.807
Meaningfulness	36.93	7.99	14	54	−0.41	0.08	0.98	0.266
Sense of coherence	120.37	21.02	54	169	−0.19	0.50	0.99	0.751
Perceived stress	20.47	7.11	1	36	−0.07	−0.48	0.98	0.248
Positive mental attitude	20.09	3.95	10	30	−0.09	0.01	0.99	0.463
Preventive behavior	19.27	5.06	6	29	−0.24	−0.33	0.98	0.199
Correct eating habits	17.88	4.78	8	27	0.03	−0.89	0.97	0.038
Health practices	18.31	4.40	6	27	0.06	−0.29	0.98	0.062
Health behaviors	75.55	14.41	31	104	−0.38	−0.12	0.98	0.187
Acceptance of illness	27.65	7.22	10	40	−0.43	−0.60	0.97	0.011

M—mean value; SD—standard deviation; min—minimum value; max—maximum value; S—skewness; K—kurtosis; S-W—Shapiro–Wilk test of normality; *p*—statistical significance.

**Table 2:** Correlation coefficients between interval variables.

Variables	1.	2.	3.	4.	5.	6.	7.	8.	9.	10.	11.	12.
1. Age	--											
2. Satisfaction with life	0.086	--										
3. Comprehensibility	0.219 *	0.415 **	--									
4. Manageability	0.190	0.374 **	0.651 **	--								
5. Meaningfulness	0.120	0.469 **	0.600 **	0.585 **	--							
6. Sense of coherence	0.206 *	0.487 **	0.878 **	0.861 **	0.843 **	--						
7. Perceived stress	−0.046	−0.520 **	−0.483 **	−0.523 **	−0.493 **	−0.579 **	--					
8. Positive mental attitude	0.256 *	0.484 **	0.430 **	0.407 **	0.540 **	0.532 **	−0.566 **	--				
9. Preventive behavior	0.221 *	0.218 *	0.198 *	0.064	0.372 **	0.246 *	−0.294 **	0.564 **	--			
10. Correct eating habits	0.247 *	0.232 *	0.379 **	0.210 *	0.344 **	0.363 **	−0.299 **	0.386 **	0.580 **	--		
11. Health practices	0.253 *	0.246 *	0.290 **	0.126	0.201 *	0.242 *	−0.300 **	0.448 **	0.499 **	0.501 **	--	
12. Health behaviors	0.307 **	0.362 **	0.402 **	0.242 *	0.454 **	0.427 **	−0.450 **	0.737 **	0.851 **	0.795 **	0.770 **	--
13. Acceptance of illness	−0.008	0.165	0.194	0.175	0.146	0.200 *	−0.268 **	0.070	0.035	0.127	0.069	0.094

* *p* < 0.05; ** *p* < 0.01.

**Table 3:** Comprehensibility, manageability, and meaningfulness as predictors of perceived stress, health behaviors, acceptance of illness, and satisfaction with life.

Dependent Variable	Predictor	*Beta*	*p*	*VIF*	*R* ^2^
Perceived	Comprehensibility	−0.17	0.146	20.02	0.317
stress	Manageability	−0.29	0.014	10.94	
	Meaningfulness	−0.23	0.040	10.75	
Positive	Comprehensibility	0.10	0.415	20.02	0.340
mental	Manageability	0.06	0.593	10.94	
attitude	Meaningfulness	0.42	0.001	10.75	
Preventive	Comprehensibility	0.05	0.689	20.02	0.219
behavior	Manageability	−0.30	0.022	10.94	
	Meaningfulness	0.49	0.001	10.75	
Correct	Comprehensibility	0.30	0.023	20.02	0.206
eating	Manageability	−0.16	0.222	10.94	
habits	Meaningfulness	0.23	0.057	10.75	
Health	Comprehensibility	0.29	0.035	20.02	0.136
Practices	Manageability	−0.16	0.239	10.94	
	Meaningfulness	0.09	0.456	10.75	
Health	Comprehensibility	0.23	0.058	20.02	0.303
Behaviors	Manageability	−0.19	0.122	10.94	
	Meaningfulness	0.39	0.001	10.75	
Acceptance	Comprehensibility	0.14	0.325	20.02	0.045
of illness	Manageability	0.08	0.556	10.94	
	Meaningfulness	0.02	0.878	10.75	
Satisfaction	Comprehensibility	0.18	0.168	20.02	0.250
with life	Manageability	0.07	0.558	10.94	
	Meaningfulness	0.32	0.007	10.75	

*Beta*—standardized regression coefficient; *p*—statistical significance; *R*^2^—determination coefficient; *VIF*—variance inflation factor.

**Table 4:** Sense of coherence as a predictor of perceived stress, health behaviors, acceptance of illness, and satisfaction with life.

Dependent Variable	*Beta*	*p*	*R* ^2^
Perceived stress	−0.60	0.001	0.341
Positive mental attitude	0.50	0.001	0.306
Preventive behavior	0.21	0.037	0.091
Correct eating habits	0.33	0.001	0.163
Health practices	0.20	0.047	0.101
Health behaviors	0.38	0.001	0.232
Acceptance of illness	0.21	0.040	0.043
Satisfaction with life	0.49	0.001	0.237

*Beta*—standardized regression coefficient; *p*—statistical significance; *R*^2^—determination coefficient.

**Table 5:** Perceived stress and health behaviors as mediators of the relationship between sense of coherence and acceptance of illness or satisfaction with life.

Predictors	Endogenous Variables	*Beta*	*p*
Sense of coherence	Perceived stress	−0.58	<0.001
Sense of coherence	Health behaviors	0.37	<0.001
Perceived stress	Acceptance of illness	−0.23	0.052
Sense of coherence	Acceptance of illness	0.07	0.567
Perceived stress	Satisfaction with life	−0.32	0.002
Health behaviors	Satisfaction with life	0.11	0.249
Sense of coherence	Satisfaction with life	0.25	0.014

*Beta*—standardized regression coefficient; *p*—statistical significance.

## Data Availability

The original data collected to conduct this study can be made available from the authors.
